# Gastric Sensory and Motor Functions and Energy Intake in Health and Obesity—Therapeutic Implications

**DOI:** 10.3390/nu13041158

**Published:** 2021-04-01

**Authors:** Lizeth Cifuentes, Michael Camilleri, Andres Acosta

**Affiliations:** 1 Precision Medicine for Obesity Program and Clinical Enteric Neuroscience Translational and Epidemiological Research Program, Department of Medicine, Mayo Clinic, Rochester, MN 55905, USA; Cifuentes.adriana@mayo.edu; 2 Division of Gastroenterology and Hepatology, Mayo Clinic, 200 First Street SW, Rochester, MN 55905, USA; Camilleri.michael@mayo.edu

**Keywords:** food intake, gastric emptying, gastric accommodation, satiation, satiety

## Abstract

Sensory and motor functions of the stomach, including gastric emptying and accommodation, have significant effects on energy consumption and appetite. Obesity is characterized by energy imbalance; altered gastric functions, such as rapid gastric emptying and large fasting gastric volume in obesity, may result in increased food intake prior to reaching usual fullness and increased appetite. Thus, many different interventions for obesity, including different diets, anti-obesity medications, bariatric endoscopy, and surgery, alter gastric functions and gastrointestinal motility. In this review, we focus on the role of the gastric and intestinal functions in food intake, pathophysiology of obesity, and obesity management.

## 1. Introduction

Obesity is a chronic and complex disease marked by a body mass index (BMI) greater than 30 kg/m^2^ and affects almost 600 million adults worldwide [1]. Obesity complexity is related to a multi-factorial imbalance between energy intake and energy expenditure, leading to excess energy storage [2]. The mechanism of moving from fasting to food-seeking and food intake is closely controlled by homeostatic and hedonic signals that combine to influence eating behavior. This homeostatic regulation involves peripheral organs and the nervous system and is referred to as the gut–brain–adipose axis [3,4]. The behavioral background is essential for explaining normal and disorderly feeding, particularly, excessive overeating, contributing to the obesity crisis. However, the central neural mechanism integrating food reward and gastric sensory and enteroendocrine signals has been adequately reviewed elsewhere [5,6,7,8,9].

In the gut, the stomach plays a significant role in regulating hunger, satiation, and satiety [10]. The stomach can sense mechanical forces, control the rate at which calories reach the duodenum, and trigger gastrointestinal peptide secretion. These functions play an essential role in providing short-term and long-term signals to control food intake [10,11]. Alterations in gastrointestinal motility observed in patients with obesity may contribute to weight gain [12,13,14]. Therefore, interventions designed to modify gastrointestinal motility or have a secondary impact on gastric physiology are components of obesity treatment.

The present paper is a narrative review based on publications focused on physiological measurements of gastric functions and their potential contribution to obesity. In particular, to understand the effects on gastric sensory and motor functions of different obesity treatments, we considered data from case-control, cross-sectional, cohort, and randomized controlled clinical trials. We excluded data measured by paracetamol drug absorption due to the potential confounding factors. Similarly, we have assessed lifestyle interventions, such as low-calorie diets, on objective measurements of gastric emptying and compared them to subjective scales for appetite. Our assessment included Food and Drug Administration (FDA) approved medications in use for diabetes or obesity. We also examined two meta-analyses that appraised effects of bariatric and surgical procedures on gastric emptying, and we updated the literature search to December 2020.

In this review, we examined the relationship between gastric sensory and motor functions with food intake. We focused first on the regulation of these functions by endocrine and neural pathways on health and discuss the abnormalities observed in obesity. We considered the significance of these functions in the pathophysiology of obesity, the contradictory findings on this topic, and have discussed the contribution of variability in these measurements in interpretation of the data. Finally, we have addressed obesity treatment strategies that target the gastric sensory and motor functions.

## 2. Regulation of Gastric Emptying and Gastric Accommodation

The stomach accommodates, triturates, and empties food into the duodenum through coordinated motor activities within proximal and distal regions. The proximal region’s critical function is to store food, while the distal region generates the mechanical forces for trituration to a particle size <2 mm that facilitates emptying and digestion. Neural and endocrine control mechanisms mediate these functions. For example, the proximal stomach exhibits low sustained contractions responsible for the basal tone. After food reaches the stomach, the vagus nerve coordinates the change in fundic compliance (also called accommodation), which allows the stomach to form a reservoir with only a limited rise in intragastric pressure [15], thereby facilitating food ingestion [16].

The ingestion of a meal is linked to coordinated contractile events in the stomach, especially in the antrum. This series of contractile activities leads to the grinding of solids and the beginning of solid emptying. Moreover, there is a positive relationship between the rate of antral contractions and the emptying of solids [17,18].

When food reaches the stomach, the body and antrum create phasic contractions that are activated by the pacemaker apparatus in the wall of the stomach consisting of interstitial cells of Cajal (ICC) and fibroblast-like cells expressing platelet-derived growth factor-alpha (PDGFR *α*) receptors [19,20].

Phasic contractions or peristaltic waves originate at the greater curve opposite to the incisura angularis and propagate toward the pylorus. The pylorus relaxes while the contractions increase in frequency and intensity in response to neurotransmitters from enteric neurons released in response to gastric distention [21]. As the pyloric sphincter remains closed, the gastric material is propelled backward where the antral contractions grind and mix the contents, resulting in a viscous pulverized material before being emptied into the duodenum [22]. The stomach empties digestible solids when the particle size has been reduced to about 2 mm or less [23].

**Figure A1:**
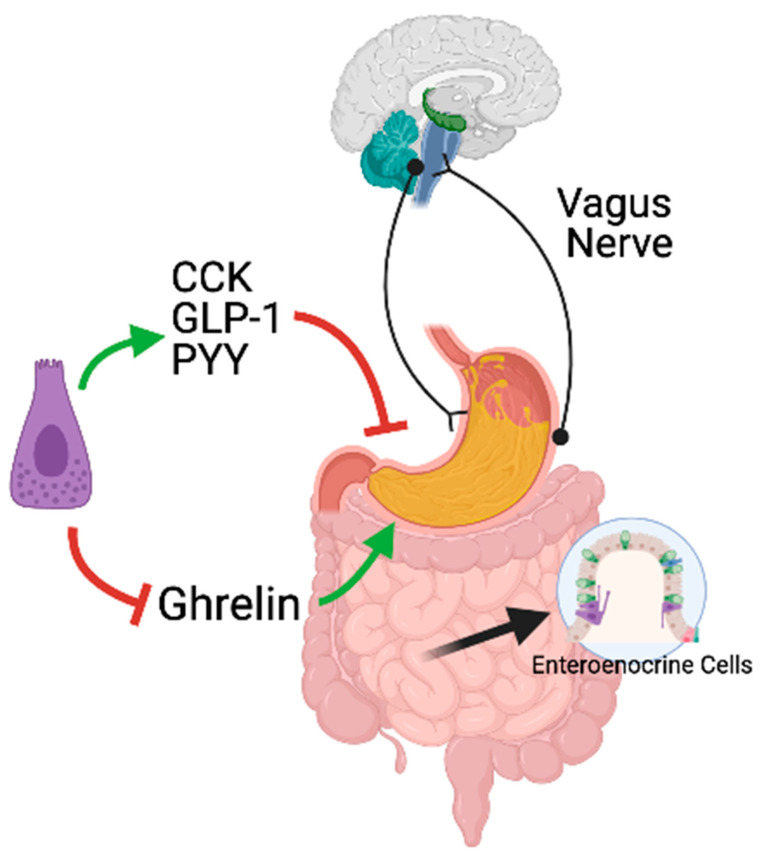
Neural and hormonal regulation of gastric emptying and gastric accommodation in response to meals. The neural regulation of gastric motor functions is commanded by the vagus nerve. The vagus nerve exerts both inhibitory and excitatory effects on the stomach. In response to nutrients, enteroendocrine cells along the gastrointestinal tract secrete CCK, GLP-1, and PYY, which inhibit gastric emptying. In response to the meal, ghrelin secretion, an orexigenic hormone known to stimulate gastric emptying, is blocked.

Thus, stomach emptying of digestible solids only starts after a lag phase required for trituration; emptying is facilitated by an increase in fundus tone, and the pyloric sphincter opens intermittently as peristaltic waves originating in the body continue to pass into the pylorus. Coordinated antro-pyloric contractions propel chyme into the duodenum [18], which relaxes and allows the well-coordinated chyme distribution. These activities are under the control of neuronal circuits and gastrointestinal hormones (Figure A1 in Appendix A). The enteroendocrine function is closely linked to gastrointestinal motor activity, which determines when and for how long the nutrients interact with enteroendocrine cells. Moreover, the release of hormones, primarily in response to nutrients, provides feedback regulation of gastric emptying.

### 2.1. Neural Regulation

Interactions between the syncytium formed by smooth muscle, ICCs and PDGFR *α* cells, the enteric system, and the central nervous system (CNS) involve multiple neurotransmitters [24]. Acetylcholine is the primary excitatory neurotransmitter, and nitric oxide and vasoactive intestinal peptides act as inhibitory neurotransmitters [25,26]. Interneurons integrate information from sensory (e.g., response to distension by food) and motor enteric neurons. The CNS provides extrinsic neural input from parasympathetic and sympathetic pathways. The sympathetic nervous system exerts a direct inhibitory influence on *α*_1_-adrenoceptors and an indirect inhibitory effect on *α*_2_-adrenoceptors. The parasympathetic nervous system exerts both excitatory and inhibitory control through the vagus nerve by activating intrinsic excitatory (e.g., cholinergic) and inhibitory (e.g., nitric oxide, vasoactive intestinal peptide, somatostatin) nerves in the stomach wall [25,27].

The vagus nerves exert both inhibitory and excitatory effects on the stomach [28]. Intraganglionic laminar endings (IGLEs) and intra-muscular arrays (IMAs) serve as sensory end-organs that activate vagal afferents. IGLEs, located between the two smooth muscle layers of the stomach, serve as tension receptors. IMAs, within the smooth muscle layer, function as stretch and length detectors [29]. Smooth muscle tone is also perceived by intrinsic nitrergic nerves and can induce fundic relaxation. The innervation from the extrinsic vagus nerve and the intrinsic nitrergic nerves also control the smooth muscle cells to mediate the postprandial accommodation response [16].

During the filling phase, accommodation maintains low intra-gastric pressure until a critical stretch level is reached and triggers vagal afferents that activate hypothalamic neurons and induce the feeling of fullness and activate tonic contractions of the fundus and peristaltic contractions in the remainder of the stomach [30].

### 2.2. Hormonal Regulation

Hormones are an effective regulatory mechanism for gastric emptying. The spectrum of hormonal actions ranges from “brake” hormones to accelerating hormones. The brake hormones include cholecystokinin (CCK); the products from pre-proglucagon, glucagon-like peptide-1 (GLP-1), glucagon-like peptide-2 (GLP-2), gastric inhibitory polypeptide (GIP), and oxyntomodulin; the polypeptide-fold family proteins peptide tyrosine-tyrosine (PYY) and pancreatic polypeptide (PP); and leptin.

In the gastrointestinal tract, CCK is released from the duodenal and jejunal mucosa by the enteroendocrine I cells in response to nutrients. Lipids, predominantly long-chain fatty acids, and proteins are the most potent stimuli [31]. CCK activates vagal afferents and thereby relaxes the proximal stomach, reduces antral contractility, and activates pyloric tone, slowing gastric emptying. CCK’s effects in gastric motility include tone reduction of the proximal stomach, suppressing antral contractions, and stimulating tonic contraction of the pylorus [32]. CCK, infused at rates to mimic postprandial plasma concentrations, slowed gastric emptying of a liquid meal by 30% and a semisolid meal by 40% [33,34]. Loxiglumide, a potent and highly specific antagonist of CCK, accelerated the gastric emptying rates of a liquid mixed meal and pure glucose meal by about 40% [35].

GLP-1 is a posttranslational product of the proglucagon gene, and it is secreted by L cells in the small intestine and colon, particularly in response to glucose and fat in the intestinal lumen [36]. In addition to a glucose-dependent action, GLP-1 can slow gastric emptying and increase postprandial gastric volume to impact satiety [37]. These actions are mediated via vagal afferent pathways and stimulation of inhibitory nitrergic myenteric neurons [38]. Accordingly, the exogenous administration of GLP-1 delays gastric emptying of liquids and solids, whereas the GLP-1 antagonist, exendin-(9–39), has been proven to accelerate gastric emptying of a mixed nutrient meal [39,40,41,42] with even an impact on glycemic responses.

Other proglucagon-derived peptides inhibit gastrointestinal motility to a lesser degree than GLP-1 [43]. Previously, GIP was considered as a component of the brake hormones, delaying gastric emptying [44]; however, physiological concentrations of GIP have failed to affect gastric emptying [45,46]. Therefore, it has not been used as a therapeutic target (e.g., in metabolic syndrome or obesity).

PYY is released from enteroendocrine L cells, both in the ileum and colon, in response to meals [47]. Endogenous PYY was associated with a decreased gastric emptying rate [48], and exogenous PYY_3–36_ resulted in a reduction in food intake without affecting gastric emptying [49]. Amylin is co-secreted with insulin from the β-cells of the pancreas, and it inhibits vagal signaling, slowing gastric emptying and promoting satiety. A decrease in the gastric emptying rate has been replicated with the amylin analog, pramlintide [50].

Leptin is released by the adipocytes around body fat stores and within the gastrointestinal system by the parietal cells of the gastric mucosa [51]. In animal studies, both intraperitoneal and intracerebral administration of leptin decreased the gastric emptying rate. Peripheral administration of leptin increased CCK release and activation of vagal cholinergic receptors, while central administration may affect the dorsal vagal complex [52,53].

Ghrelin is an orexigenic hormone produced by the cells of the gastric oxyntic mucosa and the small intestine [54]. Administration of synthetic ghrelin decreased the autonomic response to gastric distension and accelerated both gastric emptying and small bowel transit [55,56]. The infusion of the ghrelin receptor agonists, relamorelin and ulimorelin, resulted in a dose-dependent acceleration of gastric emptying. Relamorelin accelerated gastric emptying up to 17% from baseline in patients with gastroparesis without inhibiting gastric accommodation in healthy adults [57,58,59]. Recently, ulimorelin also showed a dose-dependent acceleration of gastric emptying of up to 44% from baseline in healthy adults [60].

### 2.3. Measurement of Gastric Motor Functions

A variety of tests are available to evaluate gastric motor functions; however, simultaneous assessment of gastric volume, accommodation, and emptying has only been recently reported in healthy adults, not in disease states or in response to pharmacological agents [61]. Gastric volume and accommodation can be invasively measured using an intragastric barostatically-controlled balloon or indirectly using reconstruction of transaxial images acquired with single-photon emission computed tomography (SPECT). Non-invasive imaging techniques (i.e., ultrasound, MRI, and SPECT) allow estimation of gastric volume without evaluating muscle tone [62] (Table 1).

The gold standard for measuring gastric emptying is gamma camera scintigraphy, which is a physiological, non-invasive, and objective assessment of gastric emptying. Scintigraphy comprises a radiolabeled meal, such as with ^99m^technetium, with subsequent imaging to assess emptying of solids. In 2008, the consensus statement from the American Neurogastroenterology and Motility Society and the Society of Nuclear Medicine recommended a standardized method with a low-fat, egg-white meal of 240 kcal with imaging at 0–2 and 4 h after meal ingestion [63]. Camilleri et al. published data on 314 health adults (210 females and 105 males) from an extensively validated protocol that was introduced into practice in 1991; this meal consisted of a 320 kcal, 30% fat meal with imaging at the same time points after meal ingestion [64,65]. Delayed gastric emptying is classified as >60% of the solid meal being retained at 2 h or more than 10% of the meal being left after 4 h. On the other hand, <70% retention at 30 min or <30% stomach retention at 1 h is indicative of more rapid gastric emptying [63]. Despite the protocol guidelines, several centers continue to perform sub-optimal studies (e.g., imaging for 2 h) that compromises the consistency and usefulness of the research on gastric emptying in disease states, including obesity [66].

Ultrasonography is a widely available bedside approach that quantifies changes in the cross-sectional antral area. Two-dimensional ultrasonography allows inferring gastric emptying and accommodation in various pathologies (e.g., functional dyspepsia, diabetes, gastroesophageal reflux, liver cirrhosis) and correlated antral distension with postprandial satiation in healthy subjects [67,68,69]. Three-dimensional ultrasonography reproduces the image of the total stomach and shows real-time intragastric meal distribution [70]. The volume changes provide a valid measure of gastric emptying rate in health and disease [71,72,73]. However, these techniques are difficult to standardize and are operator-dependent, or they are compromised by variation in body habitus, particularly when the proximal stomach is covered by the rib cage.

A valid alternative approach is the measurement of breath excretion of ^13^CO_2_ after ingesting a meal containing a stable isotope such as ^13^C-spirulina or ^13^C-octanoate [74]. The gastric emptying breath test constitutes an indirect, non-radioactive alternative. The subject consumes a meal with the stable isotope, and this results in a rise in expired ^13^CO_2_ that is measured [75,76].

Paracetamol absorption was proposed as a surrogate method to measure gastric emptying rate, assuming that the passage time of paracetamol through the stomach is identical to that of the meal. The plasma concentration of paracetamol was later commonly used for gastric emptying assessment [74,77]. However even when administered together with a solid meal, the paracetamol was released from the solid meal, dissolved in the liquid phase of the gastric content, and generally reflected the emptying of liquid from the stomach rather than the emptying of a solid meal. In general, most disease states are associated with normal absorption of Paris eat a mal, and therefore parameters such as the 0–5-h area under the curve of plasma paracetamol concentration is not a good reflection of the potential of a disease or pharmacological agent to modify, particularly delay, the gastric emptying of solids from the stomach. There is, moreover, a lack of consideration of pharmacokinetics in the absorption of paracetamol, which shows strong interindividual variability [78,79,80]. Using plasma levels of paracetamol for gastric emptying can contribute to incorrect conclusions. This is exemplified by the contradictory information regarding the effects of liraglutide on gastric emptying based on paracetamol absorption versus scintigraphic emptying of a mixed solid and liquid meal [81,82].

Nondigestible wireless capsules are recording devices which move through the gastrointestinal tract to detect pH, pressure, and temperature. The simultaneous intragastric measurement of pH and pressure is used to evaluate gastric emptying [83]. Barostatically-controlled balloons can calculate the gastric volume and estimate gastric accommodation. The volume is calculated based on the changes in pressure and diameter [84]. This procedure is the gold standard, but it is an invasive, non-physiologic, uncomfortable technique with relatively low reliability. However, these techniques have not been widely used in relation to obesity.

In order to assess the association between gastric motor functions and gastrointestinal symptoms or appetite sensations, a physiological test, i.e., a nutrient drink test, has been developed [85,86,87]. The test was initially designed to correlate symptoms of functional dyspepsia with maximum tolerated volume. It traditionally involved the ingestion of a nutrient drink at a constant rate of 30 mL per minute using a constant-rate perfusion pump. Participants record their sensations at 5-min intervals using a numerical scale from 0 to 5, with 0 being no symptoms, 3 corresponding to fullness sensation after a typical meal to address the volume to fullness (VTF), and 5 corresponding to the maximum tolerated volume (MTV). Nutrient intake is stopped when subjects reach a score of 5 [85]. Vijayvargiya et al. showed that, in 62 obese adults (92% females), a higher fasting gastric volume correlated with the calorie intake to reach comfortable fullness at a single meal and the VTF in the nutrient drink test [88].

## 3. Gastric Sensory and Motor Functions and Food Intake

Physiological information and external environmental cues regulate food intake and appetite. The steps in the cycle of food consumption have been determined as hunger, satiation, and satiety [89]. Hunger is defined as the desire to eat, usually associated with the duration of fasting. Satiation is the process that controls meal size and leads to meal termination, characterized by the postprandial perception of fullness or symptoms such as nausea and bloating. The number of calories consumed that is associated with these symptoms allows quantification of satiation [10,90]. Satiety denotes the intensity and duration of fullness after reaching satiation, delaying subsequent meal consumption [91]. Therefore, measurements of satiety can be based on subjective feelings or the objective duration of the period of fasting between meals.

Gastric sensory and motor functions are crucial in the regulatory process. During fasting, the stomach releases ghrelin, which induces hunger. At the initiation of the meal, stomach distention induces the sensation of satiation or fullness as originally described by Walter Cannon in 1911 in studies involving inflation of an intragastric balloon [92]. In healthy subjects, liquid preload induced gastric distention (assuming a wider antral area) and decreased both appetite and subsequent food intake [90]. On the other hand, several studies have subsequently found that increased gastric volumes are associated with a higher calorie demand for fullness [88,93]. While normal gastric accommodation facilitates food ingestion, an impaired gastric accommodation can cause gastrointestinal symptoms and gastrointestinal disorders, including functional dyspepsia [94,95].

The stomach signals sensory information for changes in volume (with or without calories) and pressure to the brain. The stomach also controls the rate of content delivered into the small intestine, thus, regulating further nutrient digestion and coordinating the enteroendocrine hormone release [96]. Gastrointestinal motility and food-activated enteroendocrine signals to the brain contribute to satiation and satiety signals. As absorption in the small intestine is highly efficient, gastric emptying is pivotal in the regulation of energy intake and nutrient absorption [97]. There is a correlation between gastric emptying of solids with the calorie intake at subsequent buffet meals and the volume to fullness. Patients with delayed gastric emptying experience fullness with lower calorie intake [90].

In addition to the neural control of food intake, emptying of nutrients from the stomach to the intestine induces neurohormonal responses. Enteroendocrine cells distributed throughout the gastrointestinal tract sense the nutrient content in the lumen and release hormones to coordinate the sensation of appetite and food consumption [10]. These enteroendocrine hormones induce local paracrine effects and distant endocrine effects, mainly in the central nervous system (CNS) [38,42,98]. The transfer of nutrients into the small intestine is essential to stimulate this endocrine control of food intake. The intestinal infusion of macronutrients can regulate appetite, with lipids being the most potent inhibitors of food intake and being highly correlated to hormonal release and gastric motor functions [99,100,101]. Therefore, the rate of nutrient delivery into the small intestine contributes to the feedback regulation of food intake.

Meal patterns and diet features contribute to the short-term control of food intake mediated by gastric motor functions. For example, four days of fasting promoted a delay in gastric emptying of liquids in patients with normal weight [102]. Timing and meal composition may influence gastric emptying. Whey protein preload has been associated with stimulation of GLP-1 release and slowing of gastric emptying [103,104,105]. This trend was sustained for four weeks after the intervention, indicating the effect of previous diet on gastric emptying [106]. Other factors such as exogenous fiber added to liquid meals or present in solids produced a small delay in gastric emptying; however, there was no impact on appetite [107,108,109]. The impact of diet history on gastric emptying highlights the challenge of standardizing results among research participants in studies of gastric functions and food intake.

Basic features such as gender, age, and BMI have been proposed as factors that influence energy intake and alter gastric emptying [110,111,112]. Gender is the major contributor to differences in gastric motor functions, with gastric emptying being around 15% slower in females and influencing the energy intake in controlled interventions [64,111]. However, the biochemical mechanisms contributing to the variance is uncertain, and research performed to determine the influence of sex hormones has failed to explain the disparity observed in women [113]. Age does not substantially change the rate of solid food emptying from the stomach, and the reported delay in liquid emptying among older participants is relatively minor and not considered clinically relevant. Still, there is an apparent decrease in caloric intake with aging and the mechanism is incompletely understood [114,115,116].

The majority of available gastric emptying trials using accurate methods have examined Caucasian patients; hence, the possible effect of ethnicity is uncertain. Data shows that some ethnicities, such as Mexican Americans, American Indians, Ethiopian refugees, and Han Chinese, have more rapid gastric emptying [117,118,119,120,121]. Physical activity may also impact gastric emptying. When compared to sedentary participants, long-distance runners had faster gastric emptying at baseline [122]. A similar pattern has been observed in individuals with obesity, where those engaging in physical exercise have demonstrated increased gastric emptying [123]. Furthermore, gastric emptying may change according to exercise intensity. High-intensity exercise may slow gastric emptying, although mild to moderate exercise has little effect or may accelerate gastric emptying [124,125].

### Gastric Sensory and Motor Functions in Obesity

The balance between energy intake and energy expenditure is a simplistic way to understand body weight regulation. An imbalance, mainly due to disruptions in food intake, is a contributor to obesity [2]. In patients with obesity, gastric volume and gastric emptying strongly correlate with hunger and fullness scores [126]. This rise in fasting gastric volumes resulted in delayed satiation, and thus a higher caloric intake is required to induce satiation, favoring weight gain [126,127]. Therefore, all processes involved in controlling gastric functions may affect body weight.

In 1975, Hunt et al. observed an accelerated transfer of calories from the stomach to the duodenum in obese patients, which was thought to lead to shorter duration of satiety [12]. Since then, gastric sensory and motor functions have been evaluated with contradictory findings, that is, both sides: rapid [14,128,129] and delayed gastric emptying have been reported [130,131,132]. These contradictory findings may be due to confounding factors such as participants’ comorbidities; concomitant medications; previous weight loss history; smoking; or study design with different criteria for participant selection, sample size, and non-standardized methods to evaluate gastric sensory and motor functions [66,133,134,135,136].

Among 328 participants, in the largest cohort reported to date, obesity was associated overall with accelerated gastric emptying, with an acceleration by 24 and 17 min for solids and 9 and 7 min for liquids in class I and class II obesity, respectively [14]. There is also evidence in normal and overweight patients that there is a relationship between weight and gastric emptying [14,137]. Furthermore, young adults with more rapid gastric emptying were shown to be more likely to gain weight [138].A larger gastric capacity has also been reported in patients with obesity based on maximum tolerated volume during a nutrient drink test or as measured by an intragastric latex balloon [139,140,141]. SPECT showed increased fasting gastric volume in participants who were overweight or obese [14,142,143]. Successful dietary weight loss was associated with a decrease in gastric volume [141].

In adult patients with obesity, baseline gastric emptying may be associated with weight loss in response to several types of interventions. The BMI change ranged from 9% for dietary interventions to 23% for Roux-Y-gastric bypass (RYGB) (additional details in next section) [82,129,144,145,146] As weight loss itself, in response to lifestyle interventions, does not seem to affect the gastric emptying rate, we may conclude that gastric emptying facilitates response to treatment [147,148,149,150,151,152]. As a result, slowing gastric emptying was suggested as one of the pathways for understanding weight loss in response to both pharmacological and endoscopic treatments. This was tested in several randomized, double-blind, placebo-controlled pilot trials with subcutaneous liraglutide and semaglutide and in a randomized, controlled trial with an intragastric balloon where retarding gastric emptying was one significant mechanism of action of those obesity treatments [90,153,154]. There is still insufficient proof of the extent of the impact of gastric emptying changes on weight loss, although it has also been shown that obese patients with accelerated gastric emptying will benefit more from specific gastric motility therapy [155].

Many syndromic and non-syndromic types of obesity provide insights into the crucial and complex role of genetics on energy balance, body weight, and the individual’s predisposition to obesity [156,157,158]. Syndromic obesity describes obesity in the clinical sense of a distinct collection of organ-specific abnormalities and additional phenotypes, such as cognitive retardation, developmental abnormalities and hyperphagia [159]. Prader–Willi syndrome is the most prevalent form of syndromic obesity; it is associated with elevated circulating ghrelin levels that lead to the observed hyperphagia [160]. Despite the hypothesis that elevated ghrelin may accelerate gastric emptying, studies have found that gastric emptying in Prader-Willi syndrome is delayed relative to reference values [161,162]. The role of gastric motor functions in other types of syndromic obesity (e.g., leptin deficiency, melanocortin 4 receptor deficiency, Bardet–Biedl syndrome, Alström syndrome) has not been assessed.

Variations in the glucagon-like peptide-1 receptor gene (*GLP1R*), the melanocortin 4 receptor gene (*MC4R*), Transcription Factor 7 Like 2 (*TCF7L2*) gene, and *CCK* gene have been studied to understand the influence of genes on gastric emptying rate and are still being studied. Several SNPs (*rs742764, rs9283907, rs2268657*, and *rs2254336)* of the GLP1R gene have been associated with statistically significant differences in gastric emptying in a pilot study [163]. The *MC4R rs17782313* CC polymorphism was associated with a 6.7% slower gastric emptying of solids at 2 h when compared with individuals with the TT genotype [164]. Cremonini et al. found an association between the *CCK* 779T > C polymorphism and slower gastric emptying rate [165]. The T allele at rs7903146 (*TCF7L2*) was non-significantly associated with faster gastric emptying [166]. Genetic variation presents a plausible area of investigation, with a specific pharmacogenetic implication as shown by Chedid et al., with GLP1R (rs6923761) being associated with more significant delay in gastric emptying T1/2 in response to liraglutide and exenatide [167].

## 4. Treatments of Obesity and Their Effects on Gastric Sensory and Motor Functions

### 4.1. Lifestyle Interventions

The first-line treatments for weight management include diet change to achieve substantial caloric deficit, increased exercise to burn calories, and behavioral treatment to improve food and physical activity patterns. Patients do not need to reach a BMI < 25 kg/m^2^ in all situations to benefit from weight loss. A sustained weight loss of less than 5% may help prevent and control diabetes, and modest weight loss of 5% to 10% has been associated with substantial improvements in cardiovascular disease risk factors [168,169]. Long-term weight maintenance is therefore essential to reducing the incidence of serious diseases. Exercise has beneficial effects on health besides weight control, but the influence of exercise-induced weight loss on gastric motor function is uncertain [170].

There are limited data available on the impact of diet and exercise-induced weight-loss on gastric motility. Table 2 details the effects of dietary interventions on gastric emptying and energy intake. The main limitations in determining the effects of dietary weight loss on gastric emptying and appetite are variation among subjects, prior dietary patterns, inclusion of patients with poorly regulated diabetes, lack of an effective matched control group, appropriate sample size, complexity in measuring gastric emptying in all settings, and lack of standardization of meals. Despite achieving significant weight-loss, most studies of lifestyle interventions have found no effect on gastric emptying after one month. However, data suggest that a rapid gastric emptying of a solid meal in patients with obesity normalizes after sustained weight loss. Changes in gastric emptying do not appear to correlate with changes in appetite.

Further studies are required to evaluate the impact of fitness programs on gastric emptying.

### 4.2. Pharmacological Treatment

Since the control of food intake is partly mediated through gastric motor functions, delaying gastric emptying may induce a decrease in calorie intake and contribute to the efficacy of weight loss in a majority of patients in the treatment of obesity. Dexfenfluramine, a discontinued anti-obesity agent, significantly slowed gastric emptying of a solid meal in obese patients following short-term therapy (5 days and 29 days) relative to placebo, leading to its effectiveness on weight loss [171].

There are five FDA-approved medications for long-term obesity care: orlistat, phentermine/topiramate, bupropion/naltrexone, and liraglutide for adults, orlistat and liraglutide for patients ≥12 years of age; and phentermine for adolescents ≥16 years [172,173,174]. Table 3 summarizes the effects of anti-obesity medications on gastric emptying in adults. The effects of orlistat on gastric emptying have been investigated in normal-weight healthy volunteers and diabetic overweight subjects where lipase inhibition resulted in a faster emptying of fats but may worsen postprandial glycemia [175,176,177,178]. However, these results have not been replicated in patients with obesity and successful weight loss after treatment [179]. In a placebo-controlled, 2-week trial of phentermine/topiramate ER, gastric emptying T1/2 accelerated by 20 min, and the drug’s effect on satiation was more pronounced, indicating a significant CNS effect on satiation resulting in the predicted weight loss [14]. GLP-1 receptor agonists are a group of GLP-1 drugs that decrease gastric emptying rate and control food intake in patients with obesity [81,98,180]. Animal models have demonstrated a decrease in food consumption with bupropion/naltrexone, but this effect and the additional improvements in gastric emptying have not been tested [181].

Six FDA-approved GLP-1 receptor agonists (i.e., exenatide, lixisenatide, liraglutide, dulaglutide, albiglutide, and semaglutide) are available. GLP-1 receptor agonists or GLP-1 analogs decrease gastric emptying rate and control food intake in patients with obesity [81,98,180]. High-dose liraglutide (3.0 mg) is the only medication in this class approved for long-term obesity treatment. Exenatide was the first to show a weight loss benefit associated with a decrease in gastric emptying [182]. A randomized clinical trial found a correlation between a delay in gastric emptying of solids following treatment with liraglutide 3.0 mg and weight loss [82]. Furthermore, gastric emptying changes may be a biomarker for a response, which may help identify patients for extended therapy [82]. Once-weekly subcutaneous semaglutide, which substantially decreased energy intake, was associated with reducing paracetamol AUC_0–1h_, strongly suggesting it may slow gastric emptying [183,184].

The short-acting GLP-1RAs (i.e., exenatide twice daily and lixisenatide) [182,185] appear to affect gastric emptying more markedly than longer-acting GLP-1RAs (i.e., exenatide once weekly, liraglutide, dulaglutide, albiglutide, semaglutide) [82,154,184,186,187]. However, it is clear that both groups slow gastric emptying, lower pre-prandial and postprandial glucose levels, and promote weight loss. Nausea and vomiting are the most frequently reported gastrointestinal adverse events for GLP-1RAs, especially for long-acting formulations, and may contribute to greater weight loss in this group [188,189]. The weight loss achieved with subcutaneous semaglutide is independent of gastrointestinal adverse events though other studies confirm that both weekly and oral semaglutide retard gastric emptying [154,189,190]. Nevertheless, weight loss associated with GLP-1 receptor agonists may be independent of gastric motor changes and related to central appetite regulation [191,192,193].

Pramlintide, an amylin analog, at 120 ug three-times-daily or 360 ug two-times-daily, resulted in long-term maintenance of weight loss and delayed gastric emptying [194]. The long-acting amylin analog, BZ043, which is under development, has demonstrated a decline in gastric emptying in animal studies [195]. PYY analogs are currently being developed based on past reports of decreased energy intake in overweight and obese people [196,197,198]. Studies in healthy humans suggest that the effects might be mediated by a decreased gastric emptying rate, among other potential mechanisms [199]. Further studies are needed to understand the role of other approved anti-obesity drugs on gastric function.

### 4.3. Endoscopic Bariatric Procedures

Endoscopic bariatric therapies (EBT) or procedures have an organ-specific target and mechanism of action [200]. Space-occupying devices are a volume-dependent weight loss therapy that encourages sensation of satiety by promoting gastric distention [141]. Fluid-filled intragastric balloons may also affect gastric emptying to facilitate weight loss [145,153,201,202]. However, most of the studies used longitudinal analysis, and only one study was a randomized controlled trial. None of the studies were able to assess possible confounders such as gender, comorbidities, baseline gastric emptying, diet history, and current use of medications.

On meta-analysis, greater changes in gastric emptying were associated with a higher percentage of total body weight lost at six months [203]. Furthermore, gastric emptying at baseline may be a valuable predictor of intolerance since increased baseline gastric retention is correlated with early balloon removal [155]. Therefore, gastric emptying can also help in selection of the appropriate patient for the procedure. Table 4 summarizes the trials evaluating EBTs and gastric emptying.

Other endoscopic restrictive techniques are likely to induce satiety. Endoscopic sleeve gastroplasty is an endoscopic gastric volume reduction technique intended to operate similarly to sleeve gastrectomy [204]. Botulinum toxin type A, a neurotoxin and inhibitor of smooth muscle contractility, has been studied because of its potential to slow gastric motility to result in earlier satiety [205]. Intragastric administration of botulinum toxin has been related to transient time and dose-dependent changes in gastric emptying [206,207]. However, the delay in gastric emptying was not associated with weight loss [203].

### 4.4. Bariatric Surgery

Table 4 summarizes the trials evaluating the effects of surgical procedures and gastric emptying.

The principal mechanisms of weight loss after bariatric surgery are malabsorption and gastric restriction. Bariatric surgery may also have beneficial effects on gut hormones, including stimulation of GLP-1 and PYY and modifying gastric motor functions [203,208,209,210,211]. These observations are indicative of the potential for other weight loss pathways to be enhanced to induce permanent weight loss.

Sleeve gastrectomy decreases gastric capacity and reduces gastric accommodation, leading to high gastric intraluminal pressure [212]. Changes in the total gastric volume, as well as gut hormones can also affect gastric emptying [213]. In a meta-analysis including 233 patients, sleeve gastrectomy was associated with a mean acceleration of gastric emptying T/2 of 29.2 min after three months. However, no significant association was found between weight loss at 1-year post-sleeve gastrectomy and gastric emptying [203,214]. Sleeve gastrectomy reduced fasting ghrelin and increased postprandial GLP-1 and PYY. These hormonal changes may impact appetite and reduce food intake [211,215].

RYGB decreased gastric capacity and accelerated the transfer of nutrients as larger particles to the distal small intestine [216]. Additionally, variations in the emptying rate varied between meals where solid emptying was slower and liquid emptying was faster following gastric bypass surgery [149]. A changes in gastric volume may be the primary determinant of weight loss, but it does not adequately explain the impact on the reward system [211,217]. Studies indicate that gastric emptying is faster following treatment; however, there is a lack of data in longitudinal studies [150,151,203,218]. On the other hand, emptying of solids from the pouch immediately after surgery was associated with increased weight loss [218,219].

Loss of normal accommodation in the gastric remnant and accelerated gastric emptying are likely to play important roles in the effects of RYGB on food intake and glycemic control. Bariatric-surgery procedures have shown, especially in the first months after surgery, to substantially increase GLP-1 secretion, which regulates gastric emptying and is related to the improved meal-related glycemia [220]. These effects might be triggered by directly transferring food to the distal small intestine with higher densities of neuroendocrine L-cells; however, there is still mixed evidence explaining the benefits of surgical procedures [221,222].

**Table 2:** Effect of low-calorie diets on gastric emptying, energy intake, and appetite in obesity.

Study Design/ Intervention	Methods	Effect on GI Motor Function	Weight Loss BMI, kg/m^2^	Energy Intake Kcal/Day	Appetite Sensations, VAS	Ref.
**RCT, SB in obesity (BMI 37.4 ± 4.0 kg/m^2^) (*n* = 42)** **Diet: ETEE-600 kcal (minimum1200 kcal), exercise and behavioral modification.**	Scinti-graphic GE and VAS: Pre and 1-month post-Rx	GE T_1/2_ liquids min: baseline 21.8 ± 10.1 vs.1-month 24.4 ± 8.7; ns GE solids %/h: baseline 30.3 ± 15.2 vs. 1-month 26.2 ± 15.2; ns	Baseline 37.4 ± 3.9 1-month 36.7 ± 3.9, Δ−0.8 (95% CI [−1.0, −0.5]; *p* < 0.001).	Δ−599.9 (95% CI [−885.6, −315.2]; *p* < 0.001).	Decrease in: Desire to eat AUC, Hunger AUC, and Fullness AUC	[179]
**RCT, DB in obesity (BMI 37.4 ± 4.0 kg/m^2^) (*n* = 14)** **Placebo vs. diet** **Diet: ETEE-600 kcal (minimum 1200 kcal).**	Scinti-graphic GE and VAS: Pre, 1- and 12 months post-Rx	GE T_1/2_ liquids min: baseline 25.5 ± 10.7; 1-month 19.3 ± 9.0; 12-months 21.8 ± 10.3; ns GE rate %/h solids: baseline 26.6 ± 16.3 1 month: 35.8 ± 13.8; ns 12-months 17.6 ± 13.0; *p* < 0.05 vs baseline	Baseline: 37.6 ± 3.9; 1-month: 37.2 ± 3.9; 12 months: 34.2 ± 5.4; *p* < 0.05	No effect	No effect	[179]
**Case-control study** **Obese (BMI, kg/m^2^: 48 [IQR 45.3–53.8) (*n* = 10).** **4 months of low-calorie diet (800–1000 kcal)**	Scinti-graphic GE and VAS: Pre vs. 1-month post-Rx	GE rate for solids %/min: Δ 0.80 (IQR: 0.60–1.15); *p* < 0.01	Weight loss: Δ 9% (0.7–23.66); *p* < 0.05	NA	No effect	[129]
**Case-control study** **19 Obese (mean BMI = 38.7 kg/m^2^): Diet-induced wt loss.** **Low-calorie diet (1000 kcal/day) for 8 weeks, then energy-restricted (1500 kcal/day) for 8 weeks,** **then maintenance diet for 8 weeks (ETEE286 kcal)**	Scinti-graphic GE: Pre and 24 weeks post-Rx	GE solids % at 30 min: Baseline 24.0 (95% CI (18.4, 29.5)), 24-weeks: 18.3 (95% CI (14.0, 22.6)); *p * < 0.02) GE solids AUC_0–60 min_ %min: Baseline 4903 (95% CI (4678, 5127)), 24-wks 4651 (95% CI (4404, 4897)); *p* < 0.03)	Baseline 38.7 (95% CI [37.2, 40.1]) vs. 24 weeks 33.0, Δ−14.7 (95% CI [30.9, 35.0]); *p* < 0.001)	NA	NA	[223]
**Cross-sectional study** **8 Obese (Weight: 148.9 kg** **[IQR 81–240)** **3 to 4 weeks of a** **very-low calorie diet.**	Scintigraphic GE: Pre- vs. 1-month post-Rx	GE for liquids, min: Baseline 41.0 ± 7.8 vs. 1-month 48.5 ± 8.7; ns GE for solids, min: Baseline 93.0 ± 11.2 vs. 1-month 100.7 ± 12.6; ns	Weight loss (mean 8.3 kg)	NA	NA	[147]

Abbreviations: AUC: area under the curve; BMI: body mass index; CI: confidence interval; DB double blind; ETEE estimated total energy expenditure; GE: gastric emptying; MIN: minutes; NA: not analyzed; ns: non-significant; VAS: visual analog scale; RCT randomized controlled trial; SB single blind.

**Table 3:** Effect of anti-obesity medications on gastric emptying and energy intake.

Study Design/ Intervention	Methods	Effect on GI Motor Function	Δ Weight, kg	Energy Intake Ad Libitum meal	Ref.
**PC, DB, RCT.** **20 obese (BMI 33.9 ± 1 kg/m^2^) with accelerated GE at baseline** **Exenatide SQ, 5 μg BID (*n* = 10) or placebo (*n* = 10)**	Scintigraphic GE test: Pre vs. 1-month post-Rx	GE % 1 h: Exenatide 12.4% (IQR 8–18.5) vs. placebo 38.2% (IQR 26.6–42.1); *p* < 0.001 GE T_1/2_, min: Exenatide 187 (IQR 141–240) vs. placebo 86 (IQR 73–125); *p* < 0.001	Exenatide −0.95 (IQR −0.7–2.1) vs. placebo 0.55 (IQR 0.3–2.1); *p =* 0.23	No effect	[182]
**RCT** **Healthy participants (BMI 29.6 ± 0.6 kg/ m^2^ vs. 29.5 ± 1.0 kg/m^2^)** **Exenatide 2.0 mg SQ weekly (*n* = 16) vs. placebo (*n* = 16)**	Scintigraphic GE test: Pre vs. 2-months post-Rx	GE for solids AUC_0–120min_: Exenatide slowed GE vs. placebo; *p* = 0.046 GE for liquids AUC_0–120min_: Exenatide slowed GE vs. placebo; *p* = 0.01	Exenatide −2.1 ± 0.5 vs. placebo 0.2 ± 0.5; *p* = 0.001	NA	[186]
**PC, DB, RCT. 40 obese** **(BMI: 34.6 kg/m^2^ vs. 37.2 kg/m^2^)** **Liraglutide group (*n* = 19) vs. placebo (*n* = 21)** **Liraglutide or placebo dose: 0.6 mg increments to 3.0 mg daily.**	Scintigraphic GE test and VAS: Pre vs. 16-weeks post-Rx	GE T_1/2_16 weeks, min: Liraglutide 142 (IQR 120–177) vs. placebo 113 min (IQR 101–133); ns GE T_1/2_16 weeks vs baseline, min: Liraglutide 30.5 (IQR -11–54) vs. placebo 1 min (IQR −19–7); *p* = 0.025	Liraglutide 5.3 (IQR 5.2–6.8) vs. placebo 2.5 (IQR 0.1–4.2); *p* = 0.0009	No effect	[82,224]
**OL, three-arm, RCT** **142 Participants with T2DM (BMI 30.8 ± 0.34 kg/m^2^)** **Rx + insulin glargine for 8 wks:** **a) Lixisenatide 20 μg SQ daily** **b) Liraglutide 1.2 mg SQ daily** **c) Liraglutide 1.8 mg SQ daily**	^13^C-sodium-octanoic acid GE test: Pre vs. 2-months post-Rx	GE T_1/2_8 weeks vs. baseline, min: Lixisenatide 20 μg 453.6 ± 58.2; *p* < 0.001 Liraglutide 1.2 mg 175.3 ± 58; *p* < 0.05 Liraglutide 1.8 mg 130.5 ± 60.3; *p* < 0.05	Lixisenatide 20 μg −1.6 ± 0.5; *p* < 0.05 Liraglutide 1.2 mg −1.8 ± 0.5; *p* < 0.05 Liraglutide 1.8 mg −2.4 ± 0.5; *p* < 0.001	NA	[225]
**OL, parallel-group, RCT** **Participants with T2DM** **Lixisenatide of 20 µg SQ daily (*n* = 69) vs. Sitagliptin 50 mg oral daily (*n* = 67)** **Lixisenatide weekly 5 µg increments: from 10 µg to 20 µg daily. **	^13^C-sodium-octanoic acid GE test: Pre vs. 1-month post-Rx.	Lixisenatide vs. sitagliptin GE AUC_0–240min_ mean change from baseline, ng/mL: −4.8 ± 0.47 vs. 0.9 ± 0.48 (−5.8 [−7.10, −4.44]; *p* < 0.0001)	Lixisenatide −0.41 vs. sitagliptin +0.39 (descriptive statistics only)	NA	[226]
**DB, X-O, RCT** **8 obese (BMI 30.3 ± 1.0 kg/m^2^) with T2DM** **Lixisenatide 10-µg SQ for 14 days and 20-µg for additional 14 days**	^13^C-octanoate GE test: 1-month post-Rx	GE AUC_1–8h_: reduced after lixisenatide compared with after placebo; *p* = 0.048	Lixisenatide −2.4 ± 4.73 vs. placebo −1.5 ± 4.24; ns	NA	[227]
**PC, DB, RCT.** **30 patients with T2DM (BMI 32.1 ± 5.1 kg/m^2^) Lixisenatide (*n* = 19) vs. placebo (*n* = 21)** **Lixisenatide or placebo dose was double weekly from 0.5 μg until a dose of 20 μg daily was reached.**	Scintigraphic GE test: Pre- vs. 8-weeks. post-Rx	Gastric retention post-Rx AUC_0–240min_: Adjusted geometric means for lixisenatide vs. placebo 2.19 (95% CI 1.82, 2.64; *p* < 0.001)	Lixisenatide −1.20 ± 5.22 vs. placebo −1.0 ± 6.22; ns	NA	[228]
**DB, PC, RCT.** **24 obese (BMI 30.3 ± 1.0 kg/m^2^)** **Phentermine/topiramate 3.75 mg and 23 mg, respectively, for the first 5 days, and 7.5 mg and 46 mg for 10 days**	Scintigraphic GE test: Pre- vs. 2-weeks. post-Rx	Phentermine/topiramate vs. placebo GE T_1/2_ Phentermine/Topiramate vs. placebo, min: 109.0 ± 7 vs. 88 ± 7; *p* = 0.05	Phentermine/topiramate −1.42 ± 0.4 vs. placebo −0.23 ± 0.4; *p* = 0.03	Phentermine-topiramate vs. placebo Δ-260 (95% CI [−491.6, −28.3]; *p* < 0.05).	[14]

Abbreviations: AUC: area under the curve; BMI: body mass index; CI: confidence interval; DB double blind; GE: gastric emptying; IQR: inter quantile range; MIN: minutes; NA: not analyzed; ns: non-significant; OL open label; RCT randomized controlled trial; RX prescription; SB single blinded; SE: standard error; T2DM: type 2 diabetes mellitus; VAS: visual analog scale; WKS weeks; X-O cross over.

**Table 4:** Effect of bariatric endoscopy on gastric emptying and energy intake.

Study Design/ Intervention	Methods	Effect on GI Motor Function	Weight Loss	Ref.
**10 subjects (BMI 32.4 ± 1.53 kg/m^2^)** **IGB filled with 200–229 mL of air for 12 weeks.**	Scintigraphic GE test: pre- and 5-wks. post-Tx	GE T_1/2_ for solids, min: baseline 57 ± 27.8 vs. 5-weeks. Post-Tx. 67 ± 27.5; *p* < 0.05	Δ3 months—BL, kg: −2.4 ± 1.04	[229]
**15 subjects (BMI 34.4 ± 0.7 kg/m^2^)** **IGB filled with 600 mL of saline for 24 weeks.**	^13^C-octanoate GE test: pre- and 16-wks. post-Tx	GE T_1/2_ for solids, min: BL 92 ± 45 vs. 16-weeks. post-Tx 157 ± 70; *p* = 0.052	Body weight loss, %: 9.4 ± 1.8	[201]
**3 subjects (BMI 40.93 ± 8.8 kg/m^2^)** **IGB filled with 500 cc of saline for 24 weeks.**	Scintigraphic GE test: pre- and 3-months post-Tx	GE T_1/2_ for solids, min: BL 114 ± 18.5 vs.12-weeks. post-Tx 375.3 ± 207; *p* = 0.02	Δ6 months—BL, kg: −14.67 ± 4.33	[202]
**7 subjects (BMI 33.76 ± 1.78 kg/m^2^)** **IGB filled with 500 cc of saline for 24 weeks.**	Scintigraphic GE test: pre- and 3-months post-Tx	GE T_1/2_ for liquids, min: BL 38.71 ± 15.91 vs. 12-wks. 318.71 ± 168.07; *p* = 0.001	Δ6 months—BL, kg: −13.14 ± 2.5	[202]
**15 subjects (BMI 34.7 ± 3.42 kg/m^2^) vs. 14 controls (BMI 35.6 ± 2.84 kg/m^2^)** **IGB + lifestyle (1000–1500 kcal) vs. lifestyle intervention alone** **IGB filled with 550 cc of saline for 24 weeks.** **Lifestyle intervention: Diet: (1000–1500 kcal), exercise, behavioral Rx**	Scintigraphic GE test: pre- and 8-, 16-wks. post-IGB	Gastric retention at 120 min after 8 weeks, %: IGB 61.4 ± 23.2 vs. controls 25.7 ± 18; *p* = 0.003 Gastric retention at 120 min after 16 weeks, %: IGB 62.1 ± 16.4 vs. controls 18.7 ± 15.6; *p* < 0.001	Δ26 wks—BL, %TBW: IGB −14 ± 7.8 vs. controls −5.4 ± 4; *p* = 0.003	[153]
**24 subjects (BMI 35.58 ± 2.79kg/m^2^)** **IGB filled with 600 cc of saline for 24 weeks.**	Scintigraphic GE test: pre- and 2-months post-IGB	GE T_1/2_ for solids, min: BL 117.92 ± 150.23 vs. 12-weeks. post-Tx 281.48 ± 206.49; *p* = 0.004	Δ6 months—BL, kg: −17.09 ± 3.34; *p* < 0.001	[230]
**20 subjects (BMI 51.7 kg/m^2^)** **BPD-DS**	Scintigraphic GE test: 3.5y postoperatively	GE T_1/2_ for solids, min: BPD-DS 28 ± 16 vs. laboratory control (*n* = 160) 91 ± 20	Δ 3.5 years—BL BMI, kg/m^2^: 51.7 vs. 31.3 (IQR 21.8–46.3)	[231]
**16 subjects (BMI 47.8 ± 1.7 kg/m^2^)** **Gastric banding**	Scintigraphic GE test: pre- and 6-months post-GB	GE rate, %/h: BL 42 (IQR 23.3–59) vs. 24-weeks. post-Tx 38 (IQR 31–71); *ns* Fundus emptying rate, %/h: BL 59 (IQR 37–91) vs. 24-weeks. post-Tx 70 (IQR 53–89); ns	Δ6 months—BL BMI, kg/m^2^: −6.1 ± 0.66; *p* < 0.001	[232]
**33 subjects (weight 76 ± 4.0 kg)** **Gastric banding (*n* = 12) vs. controls (*n* = 11)**	Scintigraphic GE test: 12-months post-GB	GE T_1/2_ for liquids, min: GB 7 ± 3 vs. controls 15 ± 2; (*p* < 0.005) GE T_1/2_ for solids: 8 patients showed slower GE T_1/2_ for solids (147 ± 25 min) vs. controls (70 ± 7 min)	Δ6 months—BL, kg: 28 ± 3	[149]
**29 subjects** **Jejunoileal Bypass**	Scintigraphic GE test: 2- and 12-months post-surgical	GE T_60 min_, %: 2 months 70 ± 24 vs. 12 months 89 ± 7; *p* < 0.05	Δ12 months—BL, kg: 42.3 ± 10.9; *p* < 0.001	[233]
**11 subjects, BMI 46.8 kg/m^2^ (IQR 35.8–62.5)** **Laparoscopic Gastric Sleeve**	Scintigraphic GE test: pre- and 6-months post-LSG	GE T_1/2_, min: BL 94.3 ± 15.4 vs. 6 months 47.6 ± 23.2; *p* < 0.01 GE at 90 min, %: BL 49.2 ± 8.7 vs. 6 months 75.4 ± 14.9; *p* < 0.01	Δ6 months—BL, kg: 42.3 ± 10.9; *p* < 0.001	[234]
**21 subjects (BMI 45.09 ± 6.2 kg/m^2^)** **Laparoscopic Gastric Sleeve**	Scintigraphic GE test: pre- and 3-months post-LSG	GE T_1/2_, min: BL 62.39 ± 19.83 vs. 3 months 56.79 ± 18.72 (*p* = 0.36, t = −0.92, ns)	Δ3 months—BL, kg: −7.29 ± 1.87; *p* <0.001	[235]
**20 subjects underwent LSG** **(BMI 38.3 kg/m^2^ [IQR 34.5–48.3])** **vs. 18 controls** **(BMI 19.8–23.5 kg/m^2^)**	Scintigraphic GE test: 3-months post-LSG	GE T_1/2_ for liquids: control vs post-surgical, min: 34.9 ± 24.6 vs. 13.6 ± 11.9; *p* < 0.01 GE T_1/2_ for solids: control vs post-surgical, min: 78 ± 15.01 vs. 38.3 ± 18.77; *p* < 0.01	Weight lost at the first month after surgery was 11.1 ± 2.2 kg and 45.5 ± 5.2 kg in the sixth months	[236]
**23 subjects underwent LSG (BMI 40.7 ± 6.6 kg/m^2^) vs. 44 controls, 24 lean (BMI 22.2 ± 2.89 kg/m^2^) and 20 obese (BMI 37.7 ± 5.4 kg/m^2^)**	Scintigraphic GE test: 2 years post-LSG	GE T_1/2_ for solids, min: lean 72.8 ± 29.6 vs. post-surgical 52.8 ± 13.5; *p* = 0.025 GE T_1/2_ for solids, min: obese controls 73.7 ± 29.0 vs. post-surgical 52.8 ± 13.5; *p* = 0.01	Δ12 months—BL, kg: −26.80 ± 5.75; *p* < 0.001	[152]
**4 subjects underwent LSG, BMI 41.9 kg/m^2^(IQR 38–44.3)**	Scintigraphic GE test: pre- and 3 months post-LSG	GE T_1/2_, min: BL 57.5 ± 12.7 vs. 3-months 32.25 ± 17.3; *p* = 0.016 GE at 90 min, %: BL 20.5 vs 3-months 9.5; *p* = 0.073	Δ3 months—BL, kg: −7.29 ± 1.87; *p* < 0.001	[237]
**45 subjects underwent LSG (BMI 49.5 kg/ m^2^)**	Scintigraphic GE test: pre- and 3 months post-LSG	GE T_1/2_, min: BL 80.4 ± 33.1 vs. 3-months 64.3 ± 40; *p* = 0.06	Pre-surgical vs. 12 months BMI, kg/m^2^: 48.5 vs. 36.8; *p* < 0.05	[238]
**21 subjects underwent LSG, BMI 46.8 kg/ m^2^ (IQR 35.8–62.5)**	Scintigraphic GE test: pre- and 4 months post LSG	GE T_1/2_, min: BL 61.7 (IQR 37.0–94.3) vs. 4-months 49.1 (IQR 22.4–92.1); *p* < 0.05	Pre-surgical vs. 6 months BMI, kg/m^2^: 46.8 (35.8–62.5) vs. 37.4 (28.2–53.2) (*p* < 0.05)	[239]
**20 subjects underwent LSG, BMI 48.7 ± 3.3 kg/ m^2^**	Scintigraphic GE test: pre- and 1–4 weeks post-LSG for liquids.	GE T_1/2_ for liquids, min: BL 25.3 ± 4.4 vs. 1-months 11.8 ± 3.0; *p* < 0.001	Δ1 month—BL BMI, kg/m^2^: −8.20 ± 1.03; *p* < 0.001	[240]
**20 subjects underwent LSG, BMI 49.1 ± 7.1 kg/ m^2^**	Scintigraphic GE test: Pre- and 4–6 weeks post-LSG for solids	GE T_1/2_for solids, min: BL 74.9 ± 7.1 vs. 6-weeks 28.4 ± 8.3; *p* < 0.001	Δ6 weeks—BL BMI, kg/m^2^: −11.40 ± 1.86; *p* < 0.001	[240]
**20 subjects underwent LSG, dichotomize according to postprandial symptoms.** **Low symptoms score,** **BMI 45.5 ± 10.7 kg/ m^2^ (*n* = 13) vs** **high symptom score, BMI, 40.5 ± 4.5 kg/m^2^ (*n* = 7)**	Scintigraphic GE test: 2 years post-LSG.	GE T_1/2_ for liquids, min: Low symptoms 10.4 ± 2.9 vs. high symptom 10.6 ± 4.3; *p* = 0.27 GE T_1/2_ for solids, min: Low symptoms 40.6 ± 10.0 vs. high symptom 34.4 ± 9.3; *p* = 0.90	Group I Δ24 months-BL BMI, kg/m^2^: −13.00 ± 3.27; *p* < 0.05 Group II Δ24 months-BL BMI, kg/m^2^: −10.50 ± 1.37; *p* < 0.05	[241]
**20 subjects underwent LSG, BMI 33.4 ± 1.2 kg/ m^2^**	Scintigraphic GE test: pre- and 3-, 6-, 12- and 24-months post-LSG	GE T_1/2_, min: BL 38.4 ± 13 vs. 3- months 20.3 ± 7.6 vs. 6-months 20.7 ± 9.5 vs. 12- months 20.6 ± 4.4; *p* < 0.05	Δ3 months—BL BMI, kg/m^2^: −5.5 ± 1.9; *p* < 0.05	[242]
**30 subjects underwent LSG, BMI 50.96 ± 5.18 kg/ m^2^**	Scintigraphic GE test: pre- and 6- and 12-months post-LSG	GE T_1/2_, min: BL 96.5 ± 78.9 vs. 6-months 44.3 ± 21.1 vs. 12-months 36.1 ± 10.2; *p* < 0.001	Δ12 months—BL BMI, kg/m^2^: −17.28 ± 6.76; *p* < 0.05	[243]
**30 subjects underwent LSG, BMI 51.27 ± 7.20 kg/ m^2^**	Scintigraphic GE test: pre- and 6- and 12-months post-LSG	GE T_1/2_, min: BL 99.9 ± 71.4 vs. 6-months 48,1 ± 21.6 vs. 12-months 44.4 ± 15.9; *p* < 0.001	Δ12 months—BL BMI, kg/m^2^: −16.79 ± 8.35; *p* < 0.05	[243]
**50 subjects underwent LSG, BMI 44.5 ± 8.1 kg/ m^2^**	Scintigraphic GE test: pre- and 3-months post-LSG	GE T_1/2_ for liquids, min: BL 26.7 ± 23 vs. 3-months 15.2 ± 13; *p* < 0.05 GE T_1/2_ for solids, min: BL 68.7 ± 25 vs. 3-months 15.2 ± 13; *p* < 0.05	%EWL after 3 months (*n* = 26): 24.6 ± 12.1 %EWL after 3 months (*n* = 26): 25.1 ± 10.9	[244]
**38 subjects underwent LSG, 12 with** **antrum resection-2 cm from the pylorus vs. 13 with antrum preservation-5 cm from the pylorus**	Scintigraphic GE test: pre- and 2-months and 1-year post-LSG	AR pre vs. 2 months post LSG GE_60-min_ for semi-solids, %: 55.8 ± 22 vs. 69.7 ± 18; ns AR pre vs. 12 months post LSG GE_60-min_ for semi-solids, %: 55.8 ± 22 vs. 66.5 ± 21; ns AP pre vs. 2 months post LSG GE_60-min_ for semi-solids, %: 52.7 ± 24 vs. 72.8 ± 20; *p* = 0.024 AP pre vs. 12 months post LSG GE_60-min_ for semi-solids, %: 52.7 ± 24 vs. 74.2 ± 16; *p* = 0.010	AR pre vs. 12 months post-LSG BMI, kg/m^2^: 43.01 vs. 31.43 AP pre vs. 12 months post-LSG BMI, kg/m^2^: 45.3 vs. 31.88	[214]
**23 subjects underwent LSG, BMI 41.9 ± 5.3 kg/ m^2^**	Scintigraphic GE test: pre- and 3-months post-LSG	GE T_1/2_ for solids, min: BL 52.7 ± 20.5 vs. 3-months 33.6 ± 3.0; *p* < 0.001	Δ3 months—BL BMI, kg/m^2^: −7 ± 7.35; *p* < 0.001	[245]
**21 subjects underwent LSG, BMI 38.89 ± 7.55kg/ m^2^**	Scintigraphic GE test: pre- and 3-months post-LSG	GE T_1/2_ for solids, min: BL 67.1 ± 33.43 vs. 3-months 20.71 ± 12.81; *p* < 0.05	Δ3 months—BL BMI, kg/m^2^: −8 ± 9.80; *p* < 0.05	[246]
**100 subjects underwent LSG, BMI 43.43 ± 3.8 kg/ m^2^**	Scintigraphic GE test: pre- and 3-months post-LSG	Retention, %: 1 h: 64 ± 13 vs. 54.5 ± 15; *p* < 0.0001 2 h: 45 ± 12 vs. 35.5 ± 13); *p* < 0.0001 4 h: 6 ± 3 vs. 4 ± 2; *p* < 0.0001	Δ3 months—BL BMI, kg/m^2^: −8.83 ± 4.54; *p* < 0.001	[247]
**23 subjects underwent LSG, BMI 42.4 ± 5.8 kg/ m^2^**	MRI GE test: pre- and after 40% of EBW loss post-LSG	Total gastric volume, mL: BL 467 (95% CI (455, 585)) vs. post-Tx 139 (95% CI (121, 185); *p* < 0.0001) Early-phase GE, mL/min: 1.9 (95% CI (1.1, 4.0)) vs. 2.69 (95% CI [1.6, 3.4]; *p* = 0.001) Late-phase GE, mL/min: 2.5 (95% CI (2.0, 2.9)) vs. 1.4 (95% CI (1.1, 1.7); *p* = 0.001)	Δ7 months—BL BMI, kg/m^2^: −9.6 ± 7.28; *p* < 0.001	[213]
**26 subjects underwent LSG, BMI 47.5 ± 6.6 kg/ m^2^**	Scintigraphic GE test: after >20% TBWL post-LSG	GE T_1/2_ for solids, min: BL 24.4 ± 11.4 vs. post-Tx 75.80 ± 45.19; *p* < 0.001	Δ8 months—BL BMI, kg/m^2^: −12.60 ± 9.99; *p* < 0.01	[248]
**10 SG (BMI 33.4 ± 2.4 kg/ m^2^), 10 RYGB (BMI 33.5 ± 2.1 kg/ m^2^), and 10 controls (BMI 33.4 ± 1.7 kg/ m^2^)**	Scintigraphic GE test: SG vs. RYGB vs. controls	GE T_1/2_ for solids, min: RYGB 11 ± 2; SG 56 ± 11; controls 113 ± 8; *p* < 0.01	BMI loss %: SG: 60 ± 8 vs. RYGB: 61 ± 7	[249]
**17 RYGB (BMI 45.8 ± 4.7 kg/ m^2^), and 9 controls (BMI 23.5 ± 1.9 kg/ m^2^)**	Scintigraphic GE test: Between 15- and 21-months post-RYGB	Emptying of pouch or stomach (fraction of total meal x hours): Liquid marker, RYGB vs. controls: 0.19 (IQR 0.07–0.26) vs. 0.49 (IQR 0.47–0.64); *p* < 0.001 Solid marker, RYGB vs. controls: 0.45 (IQR 0.31–1.04) vs. 1.33 (IQR 1.15–1.65); *p* = 0.004	Δ18 months—BL BMI, kg/m^2^: −11.20 ± 5.32; *p* = 0.04	[250]
**10 RYGB (BMI 29.9 ± 1.9 kg/ m^2^), and 10 controls (BMI 24.3 ± 0.9 kg/ m^2^)**	Scintigraphic GE test: 5 years post-surgical	RYGB vs. controls Pouch/GE T_1/2_, min: faster un RYGB; *p* < 0.001 RYGB Sitting vs. supine position Pouch/GE T_1/2_, min: 2.5 ± 0.7 vs. 16.6 ± 5.3 min; *p* = 0.02	Δ18 months—BL BMI, kg/m^2^: −12.9 ± 3.4 kg/m^2^	[221]
**8 RYGB, and 24 controls (12 lean controls vs. 12 obese controls)**	^13^C-acetate breath test GE test: RYGB 10 weeks post-surgical	Gastric emptying in lean controls and obese controls was significantly slower vs. RYGB; *p* < 0.001)	Post-surgical BMI, kg/m^2^: 38.6 ± 1.7	[251]
**10 RYGB, divided according TBWL: poor weight loss (< 25%) (*n* = 5) vs. and Successful weight loss (> 25%) (*n* = 5)**	Scintigraphic GE test: 2 years post-surgical	Poor weight loss vs. Successful weight loss Pouch/GE T_1/2_, min: 5.1 ± 1.3 vs. 34 ± 32 (*p* = 0.12) Poor weight loss vs. Successful weight loss PER_max_, %/min: 17 ± 4.7 vs. 5.6 ± 3.4; *p* = 0.002	Poor weight loss vs. Successful weight loss pre-surgical BMI, kg/m^2^: 43 ± 4.3 vs. 45 ± 3.8 Poor weight loss vs. Successful weight loss at scintigraphy, %: 17 ± 4.1 vs. 44 ± 5.7	[219]
**94 subjects underwent surgery: 47 RYGB BMI 42.4 kg/ m^2^ (IQR 36.0–54.9) and 47 BRYGB BMI 44.3 kg/ m^2^ (IQR 21.8–52.5)**	Scintigraphic GE test: Between 6 months and 2 years post-surgical	GE T_1/2_ for solids, min: RYGB 65.9 (IQR 40.6–183.0) vs. BRYGB 79.4 (IQR 41.1–390.9); *p* = 0.031	RYGB BMI, kg/m^2^: 42.4 (IQR 36.0–54.9) vs. 30.9 (IQR 23.7–43.8) BRYGB BMI, kg/m^2^: 44.3 (IQR 37.5–60.8) vs. 29.8 (IQR 21.8–52.5)	[146]

Abbreviations: AP: antral preservation; AR: Antral resection; AUC: area under the curve; BMI: body mass index; BPD-DS: biliopancreatic diversion with duodenal switch; BRYGB: Banded Roux-en-Y Gastric Bypass; CI: confidence interval; EWL: excess weight loss; GE: gastric emptying; IQR: inter quantile range; LGS: laparoscopic gastric sleeve; MIN: minutes; ns: non-significant; PER: pouch emptying rate; RYGB: Roux-en-Y Gastric Bypass; SE: standard error; TBWL: total body weight loss.

## 5. Conclusions

The effect of the stomach on food intake regulation is mediated by gastric motor and sensory functions, the former controlled by an interconnected net of intrinsic and extrinsic neuroendocrine signals. Gastric emptying and gastric accommodation are associated with appetite sensations, satiation, and satiety signals in health. These functions have been investigated as quantitative traits in relation to the pathophysiology of obesity. Despite advances in the technologies to measure gastric functions, the interpretation and standardization of studies remain challenging, which compromises the analysis of the outcomes. The variability of gastric motor and sensory functions may be influenced but not fully explained by essential characteristics such as gender, age, and weight. In the goal of fully understanding changes in gastric emptying, these variables must be taken into consideration.

Accelerated gastric emptying frequently occurs in patients with obesity. Although the magnitude of weight gain that induces acceleration remains contentious, it is appropriate to consider that gastric emptying may be one abnormal quantitative trait in obesity. Delaying gastric emptying by medications, particularly GLP-1 agonists, is a possible successful target for weight loss; however, the extent of the delay to induce weight loss remains controversial. Changes in gastric motility achieved with weight loss diets or surgical procedures may account for improvements in the gut hormone profiles, inducing release of satiety hormones and, thereby, reducing energy intake. On the road towards more personalized management of obesity, identifying patients with this specific trait may allow prediction of those who could most benefit from medications or procedures that retard gastric emptying. Future studies in treating obesity would be enhanced by optimized controlled trials that follow adequate protocols to measure gastric motility, that is, emptying and accommodation, as well as mechanisms controlling appetite, satiation, and satiety.

## Figures and Tables

**Table 1:** Methods to measure gastric motor functions.

Method	Equipment Required	Principle
Scintigraphy	External gamma camera and isotope-labeled meal	Calculating volume content after ingestion of a isotope-labeled meal, with images obtained with a gamma camera at baseline 1, 2, and 4 h.
Ultrasonography	Ultrasound scanners	Measurement of changes in antral cross-sectional area or diameter over time.
Magnetic Resonance	MRI scanner	Measurement of gastric volume, secretion, emptying, and contractions derived from repetitive scans. Gastric meal volume is calculated by taking into account gastric secretion.
Isotope breath test	Breath collection vials and stable isotope-labeled meal	Measurement of breath excretion of ^13^CO_2_ after ingestion with a solid meal. After ingestion, it is absorbed in the proximal small intestine, metabolized by the liver, and excreted by the lungs, and results in a rise in expired ^13^CO_2_.
Drug Absorption	Plasma levels of paracetamol	Measurement of plasma concentrations, assuming that small intestine intestine absorption will reflect the gastric emptying rate.
Wireless pressure and pH capsule	Intraluminal capsule with miniaturized strain gauge and pH measurement.	The simultaneous intragastric measurement of pH and pressure is used to evaluate gastric emptying.
Barostat	Barostatically-controlled balloons.	A polyethylene balloon is inserted via the esophagus, situated in the gastric fundus in apposition with the wall, and distended until an intrabag volume of 30 mL is achieved or until respiratory variation is detected. The volume is calculated based on the changes in pressure and diameter.

## Data Availability

Not applicable.
